# Putting patients first: Frontline insights on designing and administering manufacturer patient financial support programs

**DOI:** 10.1093/ajhp/zxaf271

**Published:** 2025-10-07

**Authors:** Autumn D Zuckerman, Chelsea P Renfro, Danielle Bryan, Wendi Owens, Patrick Nichols, Brandon Markley, Elizabeth Cherry

**Affiliations:** Vanderbilt Specialty Pharmacy, Vanderbilt Health, Nashville, TN, USA; Vanderbilt Specialty Pharmacy, Vanderbilt Health, Nashville, TN, USA; Vanderbilt Specialty Pharmacy, Vanderbilt Health, Nashville, TN, USA; Vanderbilt Specialty Pharmacy, Vanderbilt Health, Nashville, TN, USA; Vanderbilt Specialty Pharmacy, Vanderbilt Health, Nashville, TN, USA; Vanderbilt Specialty Pharmacy, Vanderbilt Health, Nashville, TN, USA; Vanderbilt Specialty Pharmacy, Vanderbilt Health, Nashville, TN, USA

**Keywords:** pharmacists, health services accessibility, health expenditures, financial assistance, patient participation, pharmacies

Prescription drug spending in the United States is expected to increase by 4% to 7% through 2028.^1^ Over half of prescription drug spending by insurers is for specialty medications used to treat complex chronic conditions such as cancer, inflammatory-mediated conditions, and multiple sclerosis. Though these medications are often life altering or even lifesaving, patient out-of-pocket costs are a concern. Prescription copays above $100 have been shown to affect adherence rates, and patients with Medicare who do not receive a low-income subsidy, and therefore have higher out-of-pocket costs, are more likely to abandon therapy.^2,3^ To alleviate some of the financial burden of prescription drugs on patients, most specialty medication pharmaceutical manufacturers provide financial support for the medications they bring to market through patient assistance programs (PAPs) and copay assistance programs (collectively termed patient financial support programs henceforth).

Manufacturers’ patient financial support programs are heavily used by patients prescribed specialty medications, providing millions of dollars of assistance to patients annually.^4-10^ These programs offer a clear benefit to patients by significantly reducing patient out-of-pocket costs and have been shown to improve primary adherence, secondary adherence, and medication persistence.^11^ Specialty pharmacy teams often help patients navigate specialty medication access, including insurance coverage through prior authorizations (PAs)/appeals, if necessary, and by identifying and enrolling patients in manufacturer financial assistance programs if needed.^4,9,12,13^ Therefore, pharmacy teams should be aware of key patient financial support program design features and support patient uptake and use when needed.

Eligibility requirements and enrollment procedures for patient financial support programs vary by manufacturer and medication (Table 1). PAPs offer financial assistance to eligible patients who are uninsured or underinsured (ie, enrolled in an insurance plan but lacking coverage sufficient to make prescribed medications affordable). PAP eligibility is typically income based, and enrollment requires an application process including provider and patient documentation requirements and signatures. Once approved, free drug is typically supplied by an exclusive mail order pharmacy contracted with the manufacturer to dispense the medication. Copay assistance programs include copay cards and sometimes payment cards. Only patients with commercial insurance are eligible to receive copay assistance funds, and eligibility requirements may be based on a patient’s pharmacy benefits design. Copay assistance is applied at the point of sale—it is either billed in addition to the patient’s primary commercial insurance (copay card) or billed after insurance has been applied (payment card)—to reduce out-of-pocket expenses. Notably, these programs are not required by any governing body and their administration is largely determined by manufacturer preference.

**Table 1.** Patient Financial Support Program Descriptions

Assistance type	Program overview	Program offerings	Eligibility^a^	Enrollment	Limitations
Patient assistance programs	Free medication or financial assistance offered to eligible patients who are uninsured or underinsured	Free medication fulfillment (may be through manufacturer’s pharmacy, manufacturer-contracted pharmacy, or any pharmacy)	Typically income based Requirements not published online Many exclude underinsured patients whose plan denies coverage/plan exclusion	Paper or online; Patient documents may include tax forms, paystubs, application, signature Provider documents may include referral form, signature	None as long as patient remains eligible
Copay assistance programs	Funds applied to patient’s out-of-pocket amount after insurance coverage has been applied to reduce or eliminate costs	Copay card (applied on claim) Payment card (applied after claims process)	Commercially insured patients only Residents of US Therapy used for an FDA-approved diagnosis and minimum age Pharmacy benefits restrictions^b^	Form on manufacturer website Online portal supplied to prescribers or pharmacy staff	Typically monthly or annual maximum spend amount

Abbreviation: FDA, Food and Drug Administration.

^a^Vary by manufacturer program design and medication.

^b^Eligibility may be based on a patient’s pharmacy benefits design, such as excluding copay maximizer programs.

At the core, manufacturer patient financial support programs aim to help patients access therapies. However, in practice, the design and implementation of these programs often pose unintentional challenges counter to this goal. This article will describe the 3 primary design components of patient financial support programs (communication and engagement, patient enrollment, and assistance fund application), challenges faced by frontline pharmacy teams related to each component, and recommendations for manufacturers to consider when designing each component of a program (Figure 1). Additionally, guidance for pharmacy teams to navigate these programs and recent changes to these programs and their impact on patients and frontline staff are highlighted. Finally, strategies for manufacturer consideration during pre-launch planning and post-launch management are suggested.

**Figure 1.**
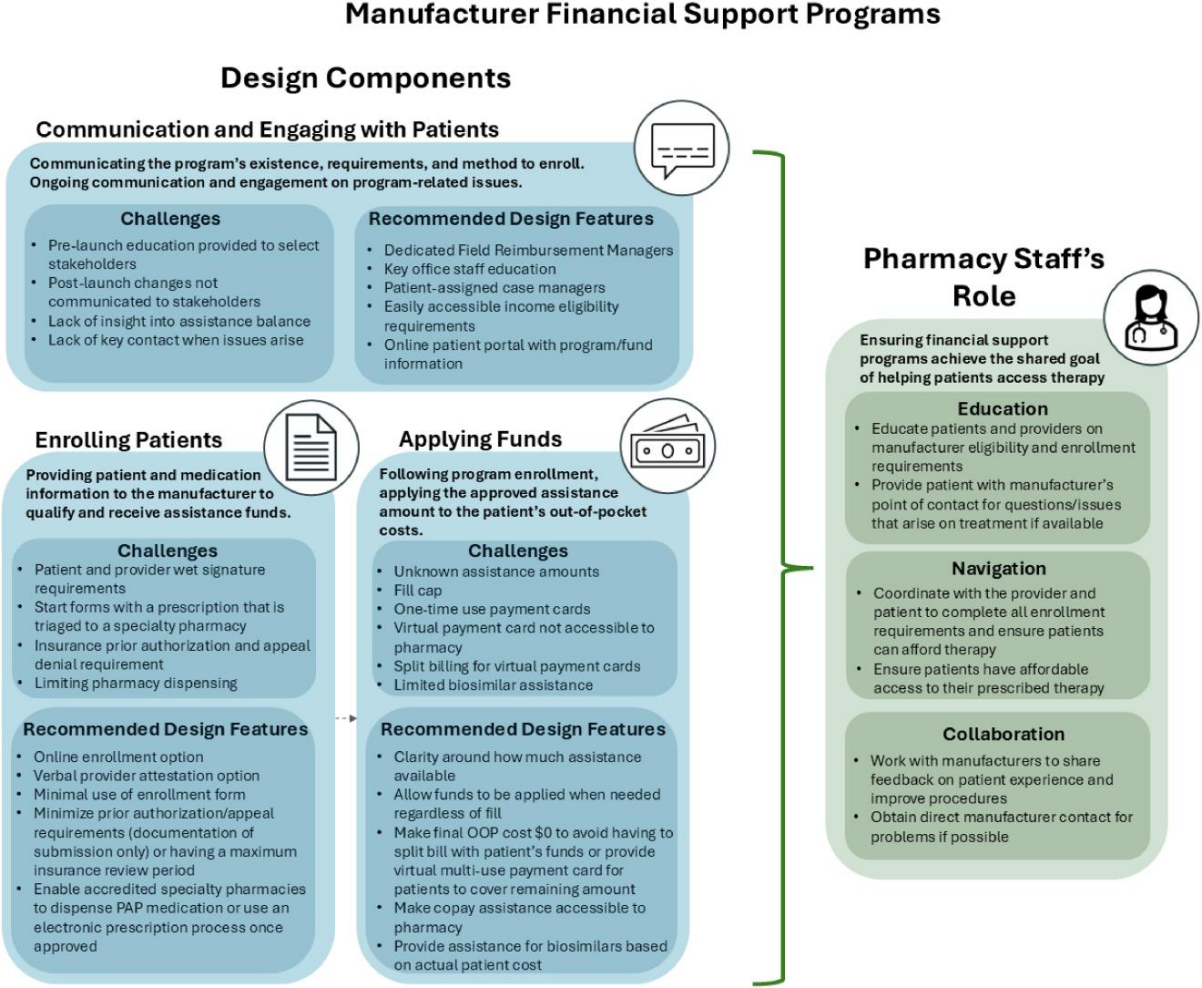
This figure depicts the 3 core program design components of patient financial support programs on the left in blue. Under each core program design component, the definition is listed in addition to challenges and recommended design features. The role of pharmacy staff is listed on the right, including education, navigation, and manufacturer collaboration. OOP indicates out-of-pocket; PAP, patient assistance program.

## Design component 1: communication and engaging with patients. *Overview*

Successful uptake of financial assistance programs requires communicating to patients and providers about their existence and design at the time of medication launch and any meaningful changes to the design of the program as they occur. Communication about the availability of financial support programs is commonly done at the time of drug launch by a field reimbursement manager (FRM) meeting with providers, pharmacy staff, or clinic staff to discuss program requirements. When changes to programs are made post launch, there is no standard method for communicating these changes to patients, providers, or pharmacy staff. There is typically minimal ongoing interaction between financial support programs for specialty medications and the patient, with the patient most often relying on the pharmacy to obtain and apply assistance funds and address any issues that arise post enrollment.

## Challenges in practice

In practice, specialty pharmacists and technicians have identified that patients are sometimes unaware of key program features such as eligibility requirements, available funds, or the method to enroll in the program. Additionally, when manufacturers change their copay assistance programs (eg, amount of available funds, claim adjudication methods, or program information location), specialty pharmacies often have difficulty finding resources detailing changes and how they should be enacted. If patients do not have access to view their available assistance funds and do not know how much to anticipate paying in the future, they may discontinue therapy if they are concerned about medication affordability. Additionally, treatment delays can occur if the pharmacy billing the assistance does not follow up-to-date procedures to enroll patients in programs. Patients may also experience issues if they change insurance and have a new deductible or out-of-pocket maximum, creating the need for additional assistance. There is often no direct contact for patient financial support programs for patients or pharmacies to reach knowledgeable agents to navigate these potential challenges in a timely manner. Rather, a “case” is created and slowly moves through an “escalation team.”

## Recommended design features

Ideally, communication regarding patient financial support programs to provider offices and pharmacies should be the responsibility of a geographically dedicated FRM. These FRMs should educate key office staff (eg, clinical pharmacists or managers, pharmacy trade relations teams, and nursing/medical assistant staff responsible for assisting patients in accessing medications) who are positioned to relay to their peers how the program details fit into clinical practice. A geographically dedicated FRM can provide the added benefit of serving as a direct contact when there are concerns or inaccuracies with the program that need to be addressed with a knowledgeable representative who can take action to address challenges. When changes are made to patient financial support programs, FRMs need to inform key office staff, pharmacy staff, and patients either by mail, electronic messaging, or phone. Copay assistance programs could also be enhanced by assigning case managers to individual patients so that when issues arise, the case manager is prepared to help troubleshoot the issue and find a resolution. To promote patient autonomy and reduce unnecessary provider/pharmacy workload, income eligibility requirements should be readily accessible to patients and pharmacy staff. Patients should also have access to their available funds and processed claims through the copay assistance program, enabling them to track their remaining assistance balance.

## Design component 2: enrolling patients into programs. *Overview*

All patient financial support programs require enrollment into the program for participation, which can occur through various methods. Common methods used include faxing paper forms, electronically submitting the form through a third-party website (eg, iAssist [AssistRx, Orlando, FL]), and enrolling the patient directly through the manufacturer’s website (often the easiest and most streamlined approach). PAP enrollment often entails submitting patient information (name, date of birth, gender, and address), income verification documents, patient consent and authorization, insurance information, provider information, the prescription, a copy of the patient’s insurance card, and provider attestation. Notably, most enrollment forms require both the patient’s and the provider’s signatures.

Copay assistance program enrollment typically only entails a patient or authorized representative providing a name, date of birth, basic demographics, pharmacy insurance billing information, and an attestation that the patient does not currently have government-funded benefits and the treatment is being used for a Food and Drug Administration (FDA)–approved diagnosis. This information is used to help confirm which plan the patient qualifies for within the copay assistance program.

Most manufacturer financial support programs use internal benefits investigation services to determine which specialty pharmacy the patient’s plan prefers to use for dispensing the specialty medication (this step is often not required), then may triage the enrollment form prescription to this specialty pharmacy.

## Challenges in practice

Patients and pharmacies often face several obstacles when enrolling patients in PAPs. Obtaining patients’ and providers’ signatures, if required, can be difficult because patients are most often enrolled in PAPs separately from a provider visit or may be seen via telehealth. In addition, obtaining the patient’s signature through electronic consent (ie, email) has also led to challenges in the timely processing of the enrollment form. A requirement to submit an insurance card if the patient has insurance can be challenging, as some patients report that they never receive a copy of their pharmacy insurance card. Requiring a prescription to be attached to the enrollment form can lead to patient and pharmacy confusion if the prescription is sent to a specialty pharmacy the patient does not plan to use. Furthermore, most specialty pharmacies no longer accept these “start-form” prescriptions as valid or legal prescriptions.

Underinsured patients may also experience a delay in treatment initiation if the PAP requires both an insurance PA and appeal denial from the provider, leaving patients without treatment while awaiting denial. Finally, not allowing specialty pharmacies to dispense the medication through the PAP and using only a paper form from the application for the prescription pose additional logistical challenges and make tracking the patient’s prescription fill challenging. Pharmacy teams or providers must identify the correct pharmacy and often lack insight into which pharmacy ultimately fills the medication.

## Recommended design features

Using manufacturer financial support programs that allow for online enrollment (ie, by the patient or the provider) and that immediately produce the copay assistance processing information (ie, ID, RxBIN, RxGroup, PCN) can lead to patients starting therapy in a timely manner. Providers should have the ability to attest to the manufacturer that patients have given them verbal permission to enroll them in the program to reduce the additional time required to re-engage the patient and obtain their signature. Ideally, enrollment forms should only be required when additional services related to the medication and provided by the manufacturer, such as refresher injection training or a new auto-injector device, are necessary to minimize the clinic workload burden. For underinsured patients, if the PAP requires documentation of PA/appeal denial, providing the submitted appeal letter to the PAP should be sufficient to demonstrate the efforts to secure insurance coverage for the medication. Another option instead of waiting on denial may be to specify a maximum review time for the insurance decision (eg, approve for PAP if no response has been received within 30 days of PA submission) to reduce treatment delays.

Accredited specialty pharmacies, particularly health-system specialty pharmacies that often coordinate medication access for patients, should be allowed to dispense medication through the PAP program. If this is not an option, once the patient is approved for PAP, an electronic prescription could be sent to the filling pharmacy for transparency and appropriate electronic health record (EHR) documentation.

## Design component 3: applying assistance funds (copay assistance programs only). *Overview*

Once the patient is aware of the copay assistance program and enrolls, funds are then applied to the out-of-pocket costs in a variety of ways based on program design and sometimes depending on the patient’s insurance plan type. These billing options include processing a copay card as a coordination of benefits with the primary insurance, use of payment cards that have funds loaded on them shortly after a claim is adjudicated to the copay card, and multistep billing procedures. Copay assistance payment cards receive funds after the copay card has been billed and are used at the point of a transaction after claims have been submitted, like a debit card. These cards, whether virtual or physical, include a card number, expiration date, and card verification code and must be handled with the same security and payment card industry compliance as a typical debit card. Manufacturers may limit the amount of assistance that can be used for each fill or may require the patient to obtain multiple payment cards to continue to receive assistance.

## Challenges in practice

When choosing how funds are applied, mandating a copay card fill cap on funds, whereby only a certain amount of funds can be used monthly or annually, can introduce challenges, as patients may have varying out-of-pocket cost requirements throughout the year, particularly when deductibles reset (most commonly in January, but this may occur anytime based on the insurance’s “plan year”). If a copay card fill cap is mandated, funds may not be available when patients are in the most financial need. Copay assistance programs that use payment cards in addition to copay cards may design these payment cards as a one-time or multiuse payment. Often one-time use cards do not count towards a patient’s insurance deductible, but multiuse cards may. The patient or pharmacy may be required to call to obtain a new one-time payment card for each fill (often monthly), and if the claim is ever reversed and readjudicated, the previously obtained virtual debit card is voided and a new one must be obtained. Therefore, multiuse cards are preferable.

Some manufacturers also limit who can obtain copay cards and payment cards. If only the patient can obtain the card (as opposed to the pharmacy obtaining it), the patient must contact the pharmacy to provide this information. For integrated health-system specialty pharmacies, the patient may attempt to enter the information through patient portals that are increasingly used for communication. However, financial data is not allowed in the EHR, and a process to delete the information must be undertaken (sometimes multiple times if the patient continues to enter this data in the portal).

When manufacturers require the final patient copay to be anything other than $0 after the copay card is applied, it can present a complex “split billing” situation if the amount of funds on the virtual payment card is unknown. The patient’s insurance is billed, then the copay card, leaving the out-of-pocket cost, which may be covered by the payment card, if available, and/or the patient. The amount of available funds that may be applied through a payment card may not be known by the pharmacy at the point of sale, and the pharmacy may be unable to bill both the payment card and the patient’s personal card at the point of sale. To avoid not overcharging the patient’s personal payment method if funds will be available on the payment card, the copay amount left after the insurance and copay card funds are applied is sometimes billed to a pharmacy invoice. Often the patient is then sent the medication to avoid delays as the pharmacy works on applying the payment card to the invoice. Sometimes the specialty pharmacy will find out after the patient has already received their medication that there were not enough funds on the payment card to reduce the patient’s responsibility to a reasonable/advertised copay. If the payment card funds are insufficient, the patient may be left with an unaffordable amount due to the pharmacy invoice. This not only makes it impossible for the specialty pharmacy to give the patient an accurate copay estimate, but it also puts both the specialty pharmacy and the patient in jeopardy if the difference between an advertised copay and actual copay is significant (possibly even several thousand dollars).

Some underinsured patients may run out of copay assistance before the end of the year or may change insurance plans and be left with a new deductible but not have copay assistance remaining. In the event of an insurance or insurance plan design change, manufacturers may “reload” a patient’s copay card, require patients to pay out of pocket and then apply for a rebate, or require patients to apply for a PAP. If applying for a PAP, patients are usually still subject to income requirements, but appeal options ideally exist. If approved for a PAP, the insurer is no longer responsible for any of the medication costs; rather, the PAP takes on the full responsibility for the medication. Often a reload of their copay card, especially after an insurance change, would alleviate the need for PAP enrollment.

Some copay assistance programs for specialty medications apply very limited funds to specialty biosimilar medications, assuming the costs for these medications should be similar to those of a typical generic. However, many payors are pricing biosimilars similarly to other specialty medications, often leaving patients with a high copay before the manufacturer copay card is applied (if available). If the amount of copay card funds that can be applied per refill has a hard cap with no workaround, the patient may be left with a copay of several thousand dollars. Often the originator biologic may be more affordable to the patient if manufacturer assistance is available, yet payors may place more biosimilars with less copay assistance available in a preferred status.

## Recommended design features

Allowing funds to be applied when they are needed, thus eliminating any fill caps, would be most useful for patients to be able to access funds when they are needed. If a fill cap is placed on the copay card, it would be helpful to also provide the patient a payment card when needed and to make known the amount of funds on the payment card so the patient can determine if it is affordable.

Copay assistance information should be accessible to both the patient and the pharmacy filling the medication to avoid the need for patients to communicate the information to the pharmacy. Ideally, copay cards could bring the final patient out-of-pocket cost to $0 for all patients, regardless of insurance plan design to avoid split billing. However, if this is not possible, it would be useful to provide a multiuse payment card that is generated after the first fill and brings the final out-of-pocket cost to the same amount for all patients.

As described above, it may be more prudent for manufacturer assistance programs to reload underinsured patients’ copay card funds to assist with payment rather than enrolling them into a PAP. Finally, biosimilar manufacturer assistance programs should reevaluate the amount of assistance being provided given the high cost of these medications.

## Recent changes and challenges to manufacturer assistance programs

Manufacturers of specialty medications are facing financial uncertainty with the introduction of price negotiation through the Inflation Reduction Act (IRA) and the increasing use of cost-shifting strategies by pharmacy benefit managers and self-funded employers, including copay maximizers, copay accumulators, and alternative funding programs.^14^ In response, many manufacturers have recently made changes to their PAPs, such as eliminating them entirely, excluding patients with commercial insurance from eligibility, or lowering the income thresholds for eligibility. These changes ultimately lead to fewer patients qualifying for and receiving manufacturer assistance funds. As a result, patients face increased financial burden and disrupted access to necessary medications. Specialty pharmacies, which most often manage patients on high-cost therapies, on the other hand, are grappling with additional administrative burdens due to the complexities of these programmatic changes.

Manufacturer copay assistance programs are increasingly performing benefits investigations to evaluate insurance plan designs to identify cost-shifting programs. Specialty pharmacies have recently seen an increase in inaccurate results of benefits investigation when performed by manufacturers or manufacturer-contracted third-party companies; this can lead to incorrectly identifying patients as being enrolled in maximizer and/or accumulator plans. These third-party companies make decisions based on partial details due to their limited access to a patient's detailed benefit information. Once these errors are identified by either the patient or the pharmacy, the burden then falls on the party that identified the inaccuracy to prove the error to the assistance program. Patients are not well versed in the terminology of these programs, and many pharmacies do not have billing specialists who have access to plan portals. The growing administrative burdens on specialty pharmacies attempting to help patients access therapy is detracting from their primary focus on patient care. Additionally, such inaccuracies delay therapy initiation, strain patient-pharmacy relationships, and could potentially lead to therapy abandonment.

## Manufacturer financial assistance and the specialty pharmacy staff’s role

Pharmacists and pharmacy technicians are knowledgeable about pharmacy benefits and financial assistance options and can communicate the benefits of assistance programs to patients. Pharmacy staff help patients access and use manufacturer financial assistance programs through education, navigation, and ongoing support (Figure 1). The pharmacy staff, informed by manufacturer liaisons, educate patients on the availability and design of financial assistance programs for specialty medications. Integrated health-system pharmacies help providers and patients navigate financial assistance programs by identifying available assistance options and assisting the patient with completing all enrollment requirements. Because of the limited communication between patients and financial assistance programs, pharmacy staff are needed to provide ongoing support as program features change or patients experience roadblocks to accessing assistance funds.

The challenges of financial assistance programs described herein often become the responsibility of the pharmacy to ensure patients can access and persist on therapy, adding significant resource demand to the pharmacy. Because pharmacists and pharmacy technicians are integral in this process and have unique insight into how program design aspects impact patient access, specialty pharmacies and manufacturers should work together to discuss and implement innovative solutions for patients that optimize resources for the manufacturer and the pharmacy. The best way to overcome these difficulties is to work directly with willing specialty pharmacy partners that use and navigate the programs on the patient’s behalf and understand the downstream effects, prior to the launch of financial support programs. Ideally, the coevaluation of the financial support programs would involve several considerations before and after a medication launch (Table 2). These learnings will ensure that the initial intent of the program is recognized and that those working directly with the patients are able to execute the program vision while minimizing the resources utilized on all fronts.

**Table 2.** Pre- and Postlaunch Program Considerations: Manufacturer and Specialty Pharmacy Collaboration

	Goal	Considerations
**Prelaunch**	Get the patient on the appropriate therapy that is fiscally manageable for ongoing use	Determine the most effective and efficient way to enroll the patient (paper vs portal, signatures required, etc) Understand the optimal approach for getting medication to the patient and factors that may impact suggested route Identify key points of contact for updates, questions, and issues as they arise Evaluate how the patient flows through the process
**Postlaunch**	Collaborate after the launch of the program as unique and unpredictable situations arise through real-world implementation	Include different perspectives on the postlaunch value of the program as barriers may vary regionally or based on pharmacy resources Ensure the initial intent of the program was recognized and those working directly with the patients are able to execute the program vision while minimizing the resources utilized on all fronts

## Conclusion

Manufacturer financial support programs play an invaluable role in helping patients access and afford high-cost specialty therapies. The design and administration of these programs can drastically impact patients’ ability to obtain treatment in a timely manner, and remain on therapy, without the anxiety of unknown costs or multiple administrative barriers to overcome. Key recommendations to communicate and engage patients within the programs, enroll patients in programs, and apply funds for patients using financial support programs have been described. Cost-shifting strategies used by insurers and concerns around the IRA price negotiation limiting revenue stream have resulted in eliminating or limiting several financial support programs. Without manufacturer financial assistance, insurers will need to reconsider the coverage of specialty medications to continue to make them accessible. By valuing the voices and experiences of pharmacy staff and patients on the frontline, manufacturers can work towards more effective financial assistance program designs, ultimately enhancing patient care and outcomes.

## Data Availability

The data underlying this article will be shared on reasonable request to the corresponding author.
